# Mixed legume–grass seeding and nitrogen fertilizer input enhance forage yield and nutritional quality by improving the soil enzyme activities in Sichuan, China

**DOI:** 10.3389/fpls.2023.1176150

**Published:** 2023-05-09

**Authors:** Muhammad Tahir, Xiao Wei, Haiping Liu, Jiayi Li, Jiqiong Zhou, Bo Kang, Dongmei Jiang, Yanhong Yan

**Affiliations:** ^1^ College of Grassland Science and Technology, Sichuan Agricultural University, Chengdu, China; ^2^ State Key Laboratory of Microbial Resources, Institute of Microbiology, Chinese Academy of Sciences, Beijing, China; ^3^ School of Life Sciences, University of Chinese Academy of Sciences, Beijing, China; ^4^ Animal Science and Technology College, Sichuan Agricultural University, Chengdu, China

**Keywords:** mixed seeding, nitrogen fertilizer, forage yield, nutritional quality, soil nutrients, soil enzymes

## Abstract

Information regarding relationships between forage yield and soil enzymes of legume–grass mixtures under nitrogen (N) fertilization can guide the decision-making during sustainable forage production. The objective was to evaluate the responses of forage yield, nutritional quality, soil nutrients, and soil enzyme activities of different cropping systems under various N inputs. Alfalfa (*Medicago sativa* L.), white clover (*Trifolium repens* L.), orchardgrass (*Dactylis glomerata* L.), and tall fescue (*Festuca arundinacea* Schreb.) were grown in monocultures and mixtures (A1: alfalfa, orchardgrass, and tall fescue; A2: alfalfa, white clover, orchardgrass, and tall fescue) under three N inputs (N1: 150 kg ha^−1^; N2, 300 kg ha^−1^; and N3: 450 kg ha^−1^) in a split plot arrangement. The results highlight that A1 mixture under N2 input had a greater forage yield of 13.88 t ha^−1^ year^−1^ than the other N inputs, whereas A2 mixture under N3 input had a greater forage of 14.39 t ha^−1^ year^−1^ than N1 input, but it was not substantially greater than N2 input (13.80 t ha^−1^ year^−1^). The crude protein (CP) content of grass monocultures and mixtures significantly (*P* < 0.05) increased with an increase in the rate of N input, and A1 and A2 mixtures under N3 input had a greater CP content of 18.91% and 18.94% dry matter, respectively, than those of grass monocultures under various N inputs. The A1 mixture under N2 and N3 inputs had a substantially greater (*P* < 0.05) ammonium N content of 16.01 and 16.75 mg kg^−1^, respectively, whereas A2 mixture under N3 had a greater nitrate N content of 4.20 mg kg^−1^ than the other cropping systems under various N inputs. The A1 and A2 mixtures under N2 input had a substantial higher (*P* < 0.05) urease enzyme activity of 0.39 and 0.39 mg g^−1^ 24 h^−1^ and hydroxylamine oxidoreductase enzyme activity of 0.45 and 0.46 mg g^−1^ 5 h^−1^, respectively, than the other cropping systems under various N inputs. Taken together, growing legume–grass mixtures under N2 input is cost-effective, sustainable, and eco-friendly, which provide greater forage yield and improved nutritional quality by the better utilization of resources.

## 1 Introduction

The growing world population has increased the demand for animal products, and the relationship is likely to get even closer in the future. With the instant development of animal husbandry, the demand for forages has emerged frequently (Hisham et al., 2022). A consistent forage supply is critical for grass-based livestock husbandry and food security. Forage grass cultivation, a pivotal ring of the feed production industry, has obvious seasonal and regional characteristics all year around (Garcez Neto et al., 2021). The natural grasslands are the primary source of forage, but cultivating legume–grass mixtures other than their respective monocultures is more beneficial because they provide higher biomass production and balanced feed for livestock (Liu et al., 2022; Tahir et al., 2022). Meanwhile, these legume–grass mixtures could face significant challenges due to lack of soil fertility, fierce competition, and scarcity of suitable species (Liu et al., 2022).

Forage production has been tightly bound up with the environment, as it is a basic industry that requires resources including land, energy, water, and labor. The cultivation of legume–grass mixtures emerged as a practical approach for increasing the forage biomass production and nutritional quality and sustaining the soil nutrient balance with minimal environmental impact (Peeters et al., 2006). Additionally, simple mixtures of two to four species may offer the best means to provide plant diversity and limit seedling competition compared with complex ones (Foster et al., 2014). Moreover, nitrogen (N) fertilization is an essential practice for the maintenance of mixture productivity, considering that a deficiency of this nutrient is a primary factor in triggering forage degradation. Generally, N inputs increase the abundance of beneficial microbes in soil that trigger the activities of microbial enzymes for nutrient mineralization (Fan et al., 2011; Sun et al., 2015). Contrarily, the negative impacts of N inputs on the soil health have also been reported by reducing the organic matter and microbial population (Marschner et al., 2003). Therefore, much attention is needed while applying N fertilizer to soils for greater forage production as N could be lost to the environment which, in turn, will become a bottleneck problem for sustainable agriculture.

The Sichuan province of China is regarded as one of the leading producers of livestock husbandry, but forage deficit, both as quantitative and qualitative, is the main constraint on the advancement of livestock husbandry in this region (Yang et al., 2023). This shortage is usually caused by the lack of soil fertility and environmental stresses that could adversely affect crop growth and development. Therefore, farmers rely heavily on N inputs to obtain a greater forage yield to counter the increased demand of forage for livestock. It is estimated that only 47% of the N added globally to soils is converted to and harvested in product form, whereas more than 50% of N is lost to the environment, which leads to waste of forage resources, threats to biodiversity and bodies of water, and increased emissions of polluting gases (Gurgel et al., 2020). Given these facts, it is of paramount importance that the current livestock systems adopt measures that utilize this nutrient with maximum efficiency. Moreover, the optimal N input rates for growing legume–grass mixtures in Sichuan, China, are not well established. Apparently, there is an urgent need to explore the combined effects of N fertilizer inputs and mixed planting on forage production, which can not only fulfill forage needs but also, more importantly, mitigate the adverse impact on the environment.

Consequently, the current study aimed to investigate the responses of forage yield and quality, soil nutrients, and soil enzyme activities to different planting patterns and N inputs. The results of this study may provide guidance for forage production in Sichuan, China, by mixed planting and optimal N inputs that could mitigate the negative environmental impacts leading towards sustainable agricultural systems.

## 2 Materials and methods

### 2.1 Research site and plant materials

A field experiment was conducted on September 15, 2018 at Modern Agriculture Research and Development Base of Sichuan Agricultural University, Chongzhou, China (103°07′ E, 30°30′ N). The legumes alfalfa [*Medicago sativa* L. (cv. Xibuzhixing)] and white clover [*Trifolium repens* L. (cv. Ladino)] and grasses orchardgrass [*Dactylis glomerata* L. (cv. Amba)] and tall fescue [*Festuca arundinacea* Schreb. (cv. Meishijia)] were selected as forage materials.

### 2.2 Soil characteristics and weather description

The soil in the upper 20 cm of the experimental field is purple clay loam with uniform fertility and has the following properties: pH, 6.30; organic matter, 37.6 g kg^−1^; alkali hydrolyzed nitrogen, 135.70 mg kg^−1^; total nitrogen, 1.81 g kg^−1^; available phosphorous, 10.20 mg kg^−1^; and available potassium, 101.10 mg kg^-1^. The climate of the experimental site is subtropical monsoon humid with an annual average temperature of 15.9°C, rainfall of 1,012.4 mm, and sunlight of 1,161.5 h.

### 2.3 Experiment design and treatment information

The field experiment was carried out in a split plot arrangement with three biological replications. A total of two legume–grass mixtures (A1: alfalfa, tall fescue, orchardgrass; A2: alfalfa, red clover, tall fescue, orchardgrass) and four monocultures (alfalfa, red clover, tall fescue, and orchardgrass) were planted in a net plot size of 5 m × 3 m under three pure N inputs (N1: 150 kg ha^−1^, N2, 300 kg ha^−1^, and N3: 450 kg ha^−1^). The main plots included three N levels, while the subplots included two legume–grass mixtures and four monocultures. The first N dose was applied at the emergence stage, while the rest of the doses were applied after each mowing. The basal inputs of P_2_O_5_ and K_2_O fertilizers were applied at 96 and 160 kg ha^−1^ year^−1^ to all plots, respectively. The seeds were handed-scattered into the soil and were covered with a thin layer of soil. Weeds that appeared in all plots were removed by hand. The seeding rates used for growing legume–grass mixtures and their corresponding monocultures are presented in **Table 1**.

**Table 1:** Seeding rates used for growing the monocultures and their mixtures (kg ha^−1^).

Plant materials	Monocultures	Mixtures^a^	
		A1	A2
Alfalfa	22.50	6.75	3.38
White clover	7.50	-	1.13
Tall fescue	37.50	13.13	13.13
Orchardgrass	15.00	5.25	5.25

a The mixtures were grown in a legume–grass ratio of 3:7.

### 2.4 Sampling and measurement

#### 2.4.1 Biomass yield

The first, second, third, and fourth cuttings for forage yield were performed during the initial flowering stage of alfalfa on March 24, May 6, July 23, and September 25 in 2019, respectively. Before harvesting, the side rows of each plot were removed, 50 cm of both sides was removed, and the area of (5–0.5 × 2) × (3–0.6) = 9.6 m^2^ was harvested to a stubble height of 5 cm. The fresh weight of the samples was recorded, and then approximately 300 g of the samples was air-dried at 65°C for 72 h up to a constant weight in an oven to estimate the dry matter (DM) content, which was later used to calculate the DM yield.

#### 2.4.2 Nutritional quality

The dried samples were ground to pass a 1-mm screen for nutritional quality analysis. The water-soluble carbohydrate (WSC) content analysis was referred to the thracenone–sulphuric acid method, while the crude protein (CP) content was measured by the Kjeldahl method (Bremner, 1996). The neutral detergent fiber (NDF) and acid detergent fiber (ADF) contents were determined according to a previously described method (Van Soest et al., 1991).

#### 2.4.3 Soil sampling

The soil samples at 15 cm depth were randomly collected from the experimental site prior to seeding and after each mowing. The five sub-samples were taken and then bulked to one sample for each replication. The soil samples were stored in cloth bags and air-dried at room temperature to a constant weight. The roots, stones, and other debris in the soil samples were removed, and the soil samples were passed through a 2 mm sieve and then stored in the laboratory until analysis.

#### 2.4.4 Soil nutrients

The pH was measured in 1/5 (w/v) aqueous extract using a pH meter. The contents of phosphorus and potassium were determined by inductively coupled plasma spectrometry after nitric-perchloric acid digestion. Soil organic matter was determined by the dilution heat method, while that of alkali hydrolyzed nitrogen was determined by the alkaline hydrolysis diffusion method (Bao, 2000). The total nitrogen was measured by the Kjeldahl method (Bremner, 1996). Soil nitrate N and ammonium N were extracted with potassium chloride (KCl, 2 mol/L), and their concentrations were measured by the flow injection method (FIA star 5000 Analyzer, FOSS, DK).

#### 2.4.5 Urease enzyme activity

The phenol-sodium hypochlorite colorimetry method was followed to measure the urease enzyme activity (Qin et al., 2016). Briefly, 10 g of dried soil was placed in a 100 ml Erlenmeyer flask, and then 2 ml of toluene, 10 ml of 10% CO(NH_2_)_2_, and 20 ml of citrate buffer were added. The samples were placed in an incubator for 24 h at 38°C. After incubation, CH_2_O was added into each sample to a constant volume of 100 ml and mixed well. The supernatant (1 ml) was taken and mixed with deionized water (9 ml), phenol (4 ml), and sodium hypochlorite solution (3 ml). The samples were placed at room temperate for 20 min. The absorbance of the samples was measured at 578 nm using a spectrophotometer.

#### 2.4.6 Hydroxylamine oxidoreductase enzyme activity

Hydroxylamine oxidoreductase (HAO) enzyme activity was determined by the ammonium ferric sulfate o-phenanthroline method. Briefly, 1 g of dried soil was taken in a test tube, and 20 mg of CaCO_3_ was added and mixed well. Then, 1 ml of 0.5% hydroxylamine hydrochloride, 1 ml of 1% glucose as hydrogen donor, and 5 ml of H_2_O were added. The samples were incubated for 5 h at 30°C in an incubator. The control sample was set without adding hydroxylamine hydrochloride and soil. After the incubation, the mixture was transferred in different test tubes, and 2 ml of alumina potassium alum saturated reagent was added. The samples were vortexed, and 1 ml of supernatant was taken and mixed with 1 ml buffer (1 mol/L CH_3_COONa and 1 mol/L CH_3_COOH), 1 ml ferric ammonium sulfate solution (0.004 mol/L), and 1 ml o-phenanthroline ethanol solution (0.01 mol/L). The color of the solution was developed for 10 min. A spectrophotometer was used to measure the absorbance at 510 nm.

#### 2.4.7 Nitrate reductase enzyme activity

The α-naphthylamine-p-aminobenzene sulfonic acid colorimetry method was followed to determine the nitrate reductase (Nar) enzyme activity (Li et al., 2014). Briefly, 1 g dried soil was taken in a test tube, and 20 mg of CaCO_3_ was added and mixed well into the former. Then, 1 ml 0.8 mmol/L 2,4-DNP solution, 1 ml 1% KNO_3_ solution, and 1 ml 1% grape poplar solution as hydrogen donor were added, and these were also mixed well. After mixing, 15 ml H_2_O was added to form the liquid seal, and the solution was incubated for 24 h at 30°C in the incubator. The control sample was set without adding 1% KNO_3_ and soil. After incubation, the solution was transferred to a different test tube, and 1 ml alumina potassium alum saturated reagent was added. Then, 1 ml of supernatant was taken and mixed with deionized water, 4 ml 0.1% α-naphthylamine solution, and 0.5% p-aminobenzenesulfonic acid. The color of the solution was developed for 15 min, and then a spectrophotometer was used to measure the absorbance at 520 nm.

#### 2.4.8 Nitrite reductase enzyme activity

Nitrite reductase (Nir) enzyme activity was also determined using the α-naphthylamine-p-aminobenzene sulfonic acid colorimetry method (Li et al., 2014). Briefly, 1 g dried soil was taken in the test tube, 20 mg CaCO_3_ was added, and these were mixed well. Then, 2 ml 0.25% NaNO_2_ solution, 1 ml 1% glucose as hydrogen donor, and 15 ml H_2_O were added. The samples were incubated for 24 h at 30°C in the incubator. The control sample was set without adding 0.25% NaNO_2_ solution and soil. After incubation, the mixture was transferred in a different test tube, and 1 ml alumina potassium alum saturated reagent was added. Then, 1 ml of supernatant was taken and mixed with deionized water, 4 ml of 0.1% α-naphthylamine solution, and 0.5% p-aminobenzenesulfonic acid. Color was developed for 15 min, and then absorbance was measured at 520 nm with a spectrophotometer.

### 2.5 Statistical analysis

All the results reported are the mean of three replicates, and the relevant data were analyzed using the SPSS software (version 28.0: IBM Corp., Armonk, NY, USA). The forage yield, nutritional quality, soil nutrients, and soil enzyme activities were analyzed using a two-way analysis of variance with Duncan’s multiple-range test. The relationships between the variables—forage yield, nutritional quality, soil nutrients, and soil enzyme activities—were determined by calculating the Pearson’s correlation coefficients and were plotted by using Origin 2022. The tables and graphics were created using Excel 2019 and GraphPad Prism 8, respectively.

## 3 Results

### 3.1 Effect of N input on the forage yield of different cropping systems

The forage yield differed substantially among the cropping systems (*P* < 0.001) and N inputs (*P* < 0.001), and the interaction between the cropping systems and N inputs was significant (*P* < 0.001) (**Table 2**). With an increase in the rate of N input, the forage yield of the alfalfa, orchardgrass, tall fescue, and A2 mixture significantly increased (*P* < 0.05), while the forage yield of white clover and A1 mixture first substantially increased and then decreased (*P* < 0.05). The alfalfa forage yield of N3 was greater than N2 and N1 by 1.3% and 7.9%, respectively; the white clover forage yield of N2 was greater than N1 and N3 by 15.2% and 15.7%, respectively; the orchardgrass forage yield of N3 was greater than N1 and N2 by 27.5% and 4.4%, respectively; the tall fescue forage yield of N3 was greater than N1 and N2 by 23.5% and 2.9%, respectively; the A1 forage yield of N2 was greater than N1 and N3 by 6.9% and 7.4%, respectively; and the A2 forage yield of N3 was greater than N1 and N2 by 25.9% and 4.1%, respectively. Taken together, the A1 mixture had a greater forage yield of 13.88 t ha^−1^ year^−1^ than the other N inputs, while that of A2 mixture under N3 had a greater forage yield of 14.39 t ha^−1^ year^−1^ than N1, but it was not substantially greater than the N2 input (13.80 t ha^−1^ year^−1^). These findings highlight that growing legume–grass mixtures under N2 input is sustainable and cost-effective, which provides greater forage yield by efficient resource utilization.

**Table 2:** Forage yield (t ha^−1^ year^−1^) of different cropping systems under various N inputs.

Cropping system	Nitrogen level			SEM	Significance	
	N1	N2	N3			
Alfalfa	12.63aA	13.54aA	13.72aAB	0.2420	Cropping system	***
White clover	8.12bC	9.57aB	8.07bD	0.2695	Nitrogen level	***
Orchardgrass	10.07bB	13.28aA	13.89aAB	0.6140	Interaction	***
Tall fescue	7.925bC	10.05aB	10.36aC	0.4154		
A1	12.92aA	13.88aA	12.85aB	0.2550		
A2	10.66bB	13.80aA	14.39aA	0.6108		
SEM	0.4868	0.4509	0.5657			

A1, mixture of alfalfa, tall fescue, and orchardgrass; A2, mixture of alfalfa, white clover, tall fescue, and orchardgrass; N1, 150 kg ha^-1^; N2, 300 kg ha^-1^; N3, 450 kg ha^-1^; SEM, standard error of the mean.

Lowercase letters represent the significant difference within the same row, while uppercase letters indicate the significant difference within the same column.

***Significance at P < 0.001.

### 3.2 Effect of N input on the nutritional quality of different cropping systems

**Figure 1:**
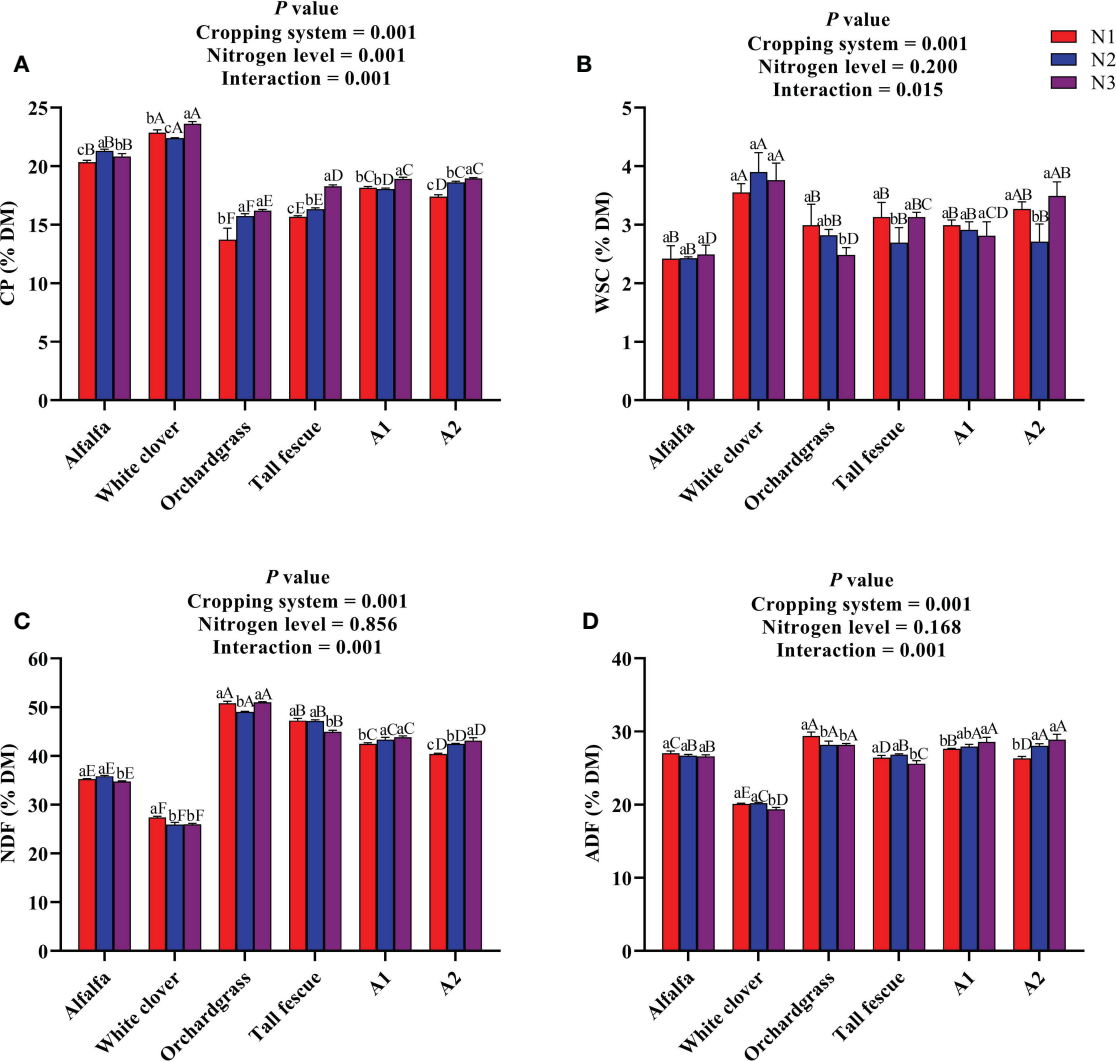
Effect of N input on the nutritional quality of different cropping systems. **(A)** Crude protein, **(B)** water-soluble carbohydrates, **(C)** neutral detergent fiber, and **(D)** acid detergent fiber. A1, mixture of alfalfa, tall fescue, and orchardgrass; A2, mixture of alfalfa, white clover, tall fescue, and orchardgrass; N1, 150 kg ha^-1^; N2, 300 kg ha^-1^; N3, 450 kg ha^-1^. The bars show the standard errors. Lowercase letters represent the significant difference within the same cropping system under various N inputs, while uppercase letters indicate the significant difference within different cropping systems under the same N input. Significance was employed at 0.05.

The nutritional quality of different cropping systems under various N inputs is presented in **Figure 1**. The cropping system and N level significantly influenced the CP content (*P* < 0.001), and the interaction among the cropping system and N level was also significant (*P* < 0.001) (**Figure 1A**). The legume monocultures had a greater CP content than the others; however, the CP content in the grass monocultures and mixtures substantially increased (*P* < 0.05) with an increase in the rate of N input. Additionally, the A1 and A2 mixtures had a greater CP content of 18.91% and 18.94% of DM, respectively, under N3 input than the grass monocultures under various N inputs. The cropping system substantially affected the WSC content (*P* < 0.001), and while the N level did not influence the WSC content, but their interaction was significant on the WSC content (*P* < 0.05) (**Figure 1B**). The white clover had a greater WSC content (3.5%–3.9% DM) followed by the A2 mixture (2.7%–3.5% DM), while alfalfa (2.4%–2.5% DM) and orchardgrass (2.5%–2.9% DM) had a lower WSC content than the other cropping systems at different N inputs. The cropping system substantially influenced the fiber contents (*P* < 0.001), while the N level had a non-significant effect, but their interaction was significant on the fiber contents (*P* < 0.001) (**Figures 1C, D**). The legume monocultures had lower NDF and ADF contents than those of grass monocultures and mixtures under various N inputs. Meanwhile, the mixtures had lower NDF (40.44%–43.86% DM) and ADF (26.32%–28.89% DM) contents at different N inputs when compared with the orchardgrass monoculture. Taken together, cultivating grasses in combination with legumes improved the nutritional quality of forages, while N fertilization did not affect the nutritional quality of forages except the CP content.

### 3.3 Effect of N input on the soil nutrients of different cropping systems

**Figure 2:**
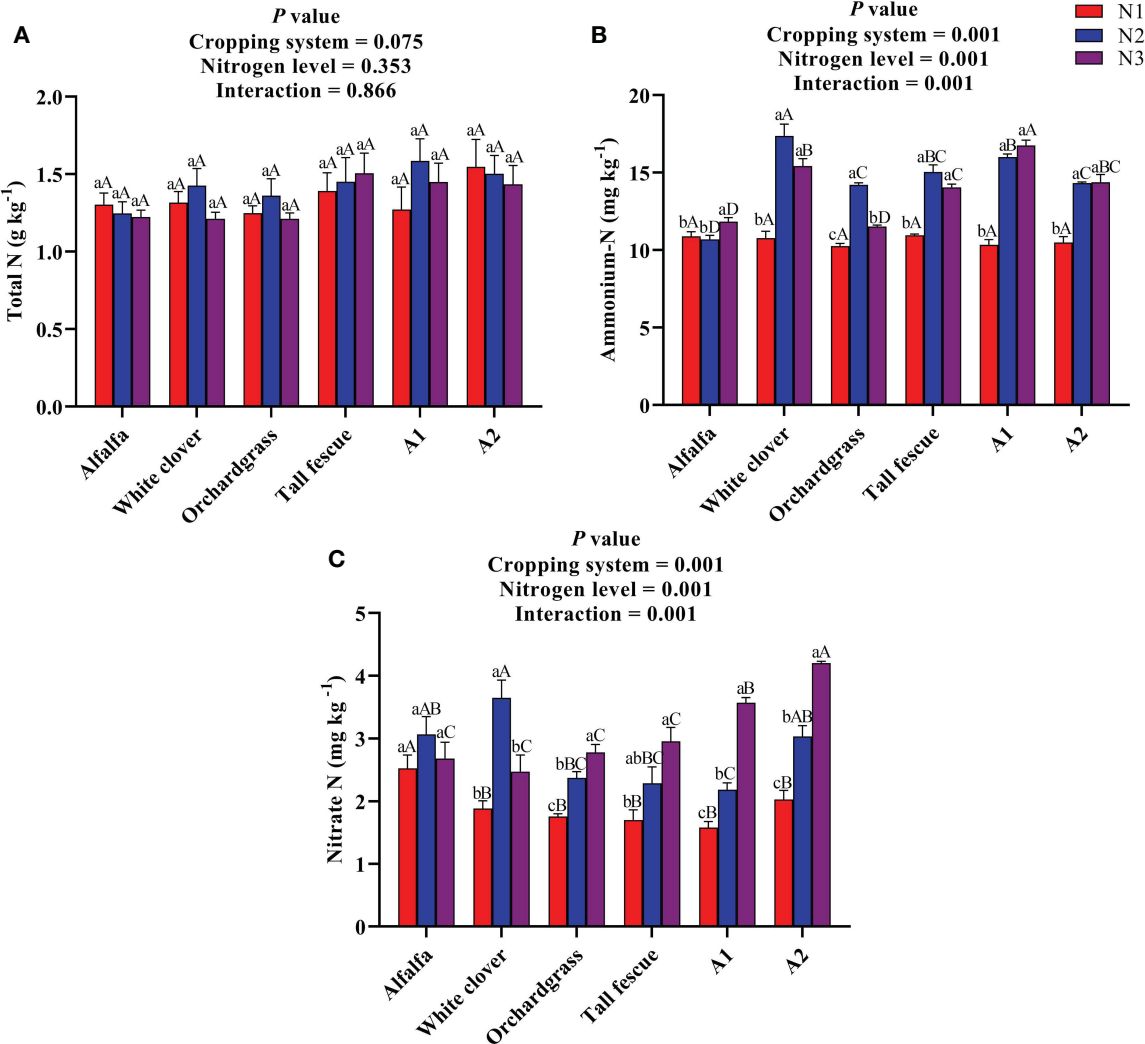
Effect of N input on the soil nutrients of different cropping systems. **(A)** Total N, **(B)** ammonium N, and **(C)** nitrate N. A1, mixture of alfalfa, tall fescue, and orchardgrass; A2, mixture of alfalfa, white clover, tall fescue, and orchardgrass; N1, 150 kg ha^-1^; N2, 300 kg ha^-1^; N3, 450 kg ha^-1^. The bars show the standard errors. Lowercase letters represent the significant difference within the same cropping system under different N inputs, while uppercase letters indicate the significant difference within different cropping systems under the same N input. Significance was employed at 0.05.

The effect of N input on the soil nutrients of the different cropping systems is shown in **Figure 2**. The total N content was neither affected by the cropping system nor the N level, and their interaction was also non-significant on total N (**Figure 2A**). The total N content of the different cropping systems under various N inputs ranged from 1.21–1.58 g kg^−1^; the total N content of the A1 mixture under N2 input (1.58 g kg^−1^) was numerically greater than those of other cropping systems under various N inputs. The ammonium N content was substantially affected by the cropping system (*P* < 0.001) and N level (*P* < 0.001), and their interaction was also significant for ammonium N content (*P* < 0.001) (**Figure 2B**). With an increase in the rate of N input, the ammonium N content of alfalfa and mixtures significantly increased (*P* < 0.05), while the ammonium N content of white clover and grass monocultures first increased and then decreased (*P* < 0.05). The A1 mixture had a greater ammonium N content of 16.01 and 16.75 mg kg^−1^ under N2 and N3 inputs than the other cropping systems (except white clover under N2 input: 17.02 mg kg^−1^) under various N inputs. The nitrate N content was significantly influenced by the cropping system (*P* < 0.001) and N level (*P* < 0.001), and their interaction was also significant for nitrate N content (*P* < 0.001) (**Figure 2C**). With an increase in the rate of N input, the nitrate N content of grass monocultures and mixtures substantially increased (*P* < 0.05), whereas the nitrate content of legume monocultures first increased and then decreased (*P* < 0.05). A1 and A2 had a greater nitrate N content of 3.6 and 4.2 mg kg^−1^ under N3 input compared with the other cropping systems under various N inputs. Taken together, the legume–grass mixed cultivation substantially increases the contents of available N in the soil when the N input rate was ≥300 kg ha^−1^.

### 3.4 Effect of N input on the soil enzyme activities of different cropping systems

**Figure 3:**
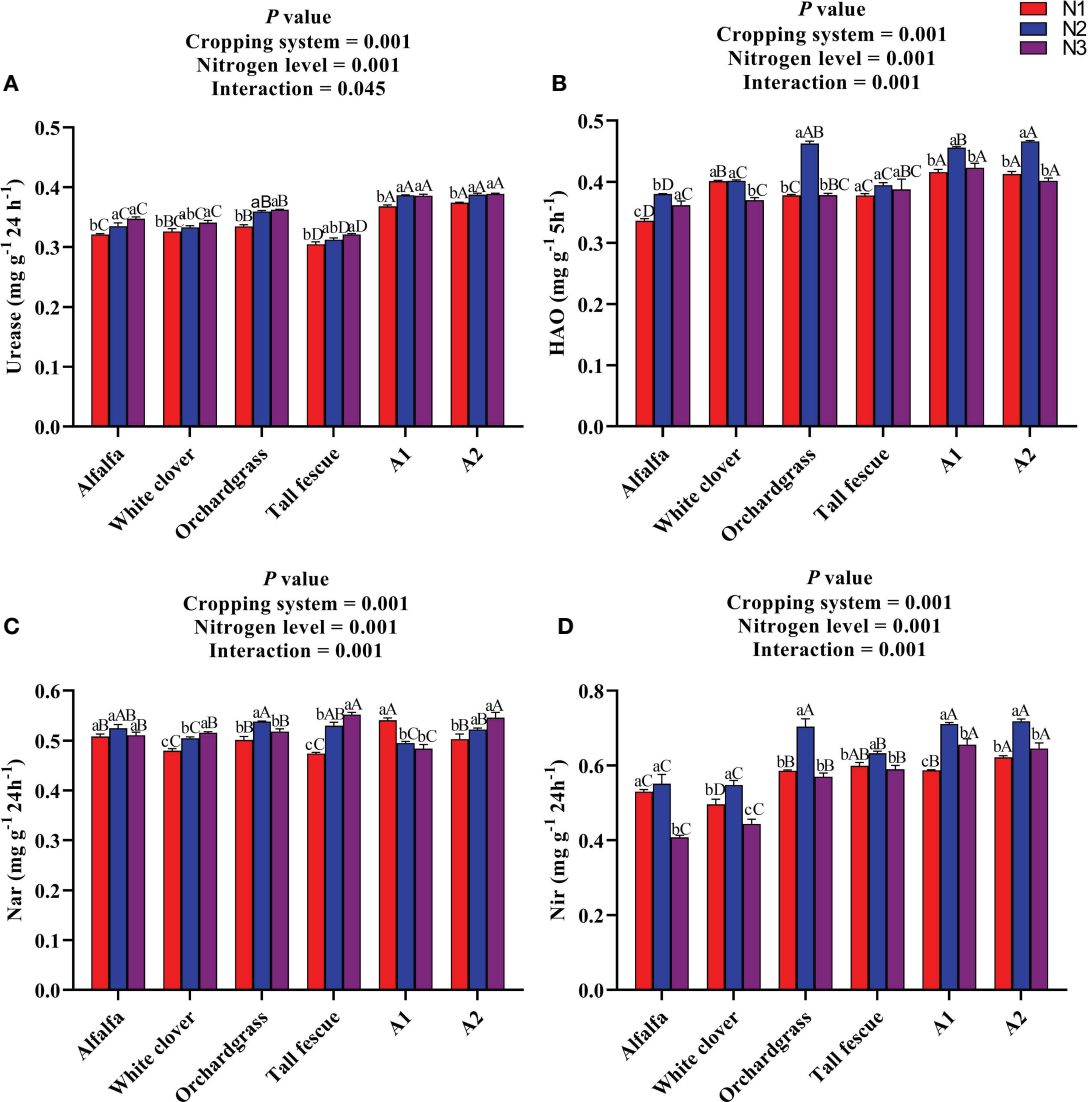
Effect of N input on the soil enzyme activities of different cropping systems. **(A)** Urease, **(B)** hydroxylamine oxidoreductase, **(C)** nitrate reductase, and **(D)** nitrite reductase. A1, mixture of alfalfa, tall fescue, and orchardgrass; A2, mixture of alfalfa, white clover, tall fescue, and orchardgrass; N1, 150 kg ha^-1^; N2, 300 kg ha^-1^; N3, 450 kg ha^-1^. The bars show the standard errors. Lowercase letters represent the significant difference within the same cropping system under different N inputs, while uppercase letters indicate the significant difference within different cropping systems under the same N input. Significance was employed at 0.05.

The influence of N input on the soil enzyme activities of different cropping systems is presented in **Figure 3**. The urease enzyme activity was substantially affected by the N level (*P* < 0.001), cropping system (*P* < 0.001), and their interaction (*P* < 0.001) (**Figure 3A**). The urease activity of all cropping systems significantly increased (*P* < 0.05) with an increase in the rate of N input, whereas no substantial difference was observed among the N2 and N3 inputs of the cropping systems. The A1 and A2 mixture had a greater urease activity of 0.39 and 0.39 mg g^−1^ 24 h^−1^ under N2 input than the other cropping systems under various N inputs. The HAO enzyme activity was significantly influenced by the N level (*P* < 0.001) and cropping system (*P* < 0.001), and their interaction was also significant for the HAO enzyme activity (*P* < 0.001) (**Figure 3B**). The HAO enzyme activity of all cropping systems, except the white clover monoculture, first increased and then decreased with an increase in the rate of N input, and it was substantially greater under N2 input compared with the other N inputs. The A2 and A1 mixtures had the comparative HAO enzyme activities of 0.46 and 0.45 mg g^−1^ 5 h^−1^ with orchardgrass HAO enzyme activity of 0.46 mg g^−1^ 5 h^−1^ under N2 input, which were greater than those of other cropping systems under various N inputs. The Nar enzyme activity differed among the cropping systems (*P* < 0.001) and N level (*P* < 0.001), and the interaction of the cropping system and N level was significant for the Nar enzyme activity (*P* < 0.001) (**Figure 3C**). The Nar enzyme activity of white clover, tall fescue, and A2 mixture significantly increased (*P* < 0.05), whereas the Nar enzyme activity of the A1 mixture substantially decreased (*P* < 0.05) with an increase in the rate of N input. The A1 mixture had a lower Nar enzyme activity of 0.48 mg g^−1^ 24 h^−1^ under N3 input than the other cropping systems under various N inputs (except tall fescue under N1 input). The Nir enzyme activity was significantly influenced by the cropping systems (*P* < 0.001) and N level (*P* < 0.001) and their interaction (*P* < 0.001) (**Figure 3D**). With an increase in the rate of N input, the Nir activity of all cropping systems increased first and then decreased. The alfalfa and white clover monoculture had lower Nir activities of 0.40 and 0.44 mg g^−1^ 24 h^−1^ under N3 input than the other cropping systems under various N inputs. Taken together, the N addition and legume–grass mixed cultivation significantly improve the urease and HAO enzyme activities which play a crucial role in releasing more available N in the soil for plant uptake.

### 3.5 Relationships between forage yield, soil nutrients, and soil enzyme activities

**Figure 4:**
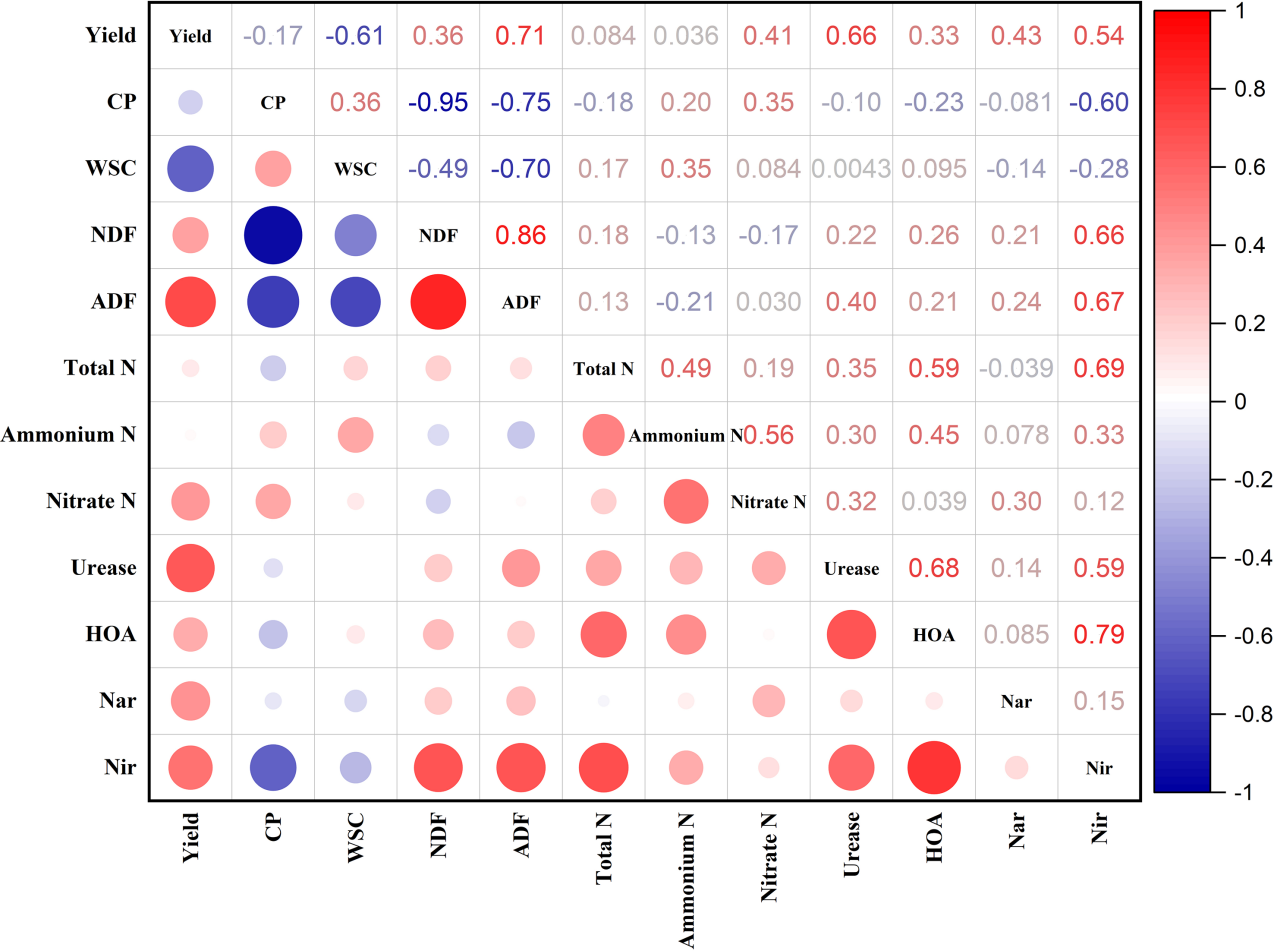
Associations among forage yield and nutritional quality, soil nutrients, and soil enzyme activities. CP, crude protein; WSC, water-soluble carbohydrates; NDF, neutral detergent fiber; ADF, acid detergent fiber; HAO, hydroxylamine oxidoreductase; Nar, nitrate reductase; and Nir, nitrite reductase.

The relationships among forage yield, nutritional quality, soil nutrients, and soil enzyme activities are presented in **Figure 4**. The forage yield was substantially positively correlated (*P* < 0.05) with soil enzyme activities, nitrate N, ADF, and ADF, while it was significantly negatively correlated (*P* < 0.05) with WSC. The CP had substantially positive relationships (*P* < 0.05) with nitrate N and WSC, while it had significantly negative correlations (*P* < 0.05) with NDF, ADF, and Nir activity. The Nir enzyme activity was significantly positively correlated (*P* < 0.05) with fiber contents, total N, ammonium N, urease, and HAO activity. The urease activity was substantially positively associated (*P* < 0.05) with soil nutrients, while the HAO activity was significantly positively correlated (*P* < 0.05) with total N, urease activity, and ammonium N.

## 4 Discussion

### 4.1 Response of soil enzyme activities of different cropping systems to N input

Soil enzyme activities are important in decomposing organic matter, recycling nutrients, and influencing microbial functions (Sinsabaugh et al., 2008). Urease is an enzyme that degrades urea and is widely regarded as an accurate predictor of N mineralization (Das and Varma, 2011). In this study, the legume–grass mixtures had greater urease enzyme activity than their corresponding monocultures, and its activity increased with an increase in the rate of N input, but there was no substantial difference among the N2 and N3 inputs. This advantageous effect of mixed sowing and N input on urease enzyme activity in soil could be attributed to an increase in microbial population as well as the release of a greater proportion of nitrogenous substances (ammonium N and nitrate N) in root exudates that can induce urease enzyme activity and become available for plant uptake. Improvement in urease enzyme activity is highly dependent on the availability of substrates like urea or ammonium-based fertilizers for nitrogen-cycling enzymes, which results in increased enzyme activity as a positive association has been reported between the substrate and urease activity (Ibrahim et al., 2020). Meanwhile, when the N input increased from N2 to N3, the urease enzyme activity did not increase significantly, which may be due to the absorption of mineral N by soil microorganisms or buildup of NH^+4^ that suppressed the urease activity (Kumari et al., 2020).

HAO is a key enzyme in the nitrification pathway, and its activity is usually dependent on the abundance and community structure of ammonia-oxidizing bacteria. In this study, the HAO enzyme activity increased first and then decreased with an increase in the rate of N input, and the legume–grass mixtures and orchardgrass had a greater HAO enzyme activity at N2 input. This could be attributed to the soil environment of legume–grass mixtures and orchardgrass which was more conducive for the growth of ammonia-oxidizing bacteria that ultimately led to an increase in HAO enzyme activity. It has been established that N addition to soil increases the abundance of ammonia-oxidizing bacteria that further improved the HAO enzyme activity, which is beneficial to enhance the available N for plant uptake (Carey et al., 2016). Meanwhile, the decrease of HAO at a higher rate of N input might be related to environmental stresses such as acidification, which influences the substrate availability and abundance of ammonia-oxidizing bacteria, leading to a lower HAO enzyme activity (Liu et al., 2018).

Nar and Nir are the key enzymes for the denitrification process in which nitrate and nitrite are reduced into NO, N_2_O, and N_2_ when oxygen is limited. This process generally causes N loss from agricultural soils and contributes toward greenhouse gas N_2_O emission (Chen et al., 2012). The Nar enzyme activity of all cropping systems except A1 is enhanced with an increase in the rate of N input, while the Nir enzyme activity first increased and then decreased with an increase in the rate of N input, and legume monocultures had a lower Nir enzyme activity at all N inputs compared with others. The previous study has reported that N addition subsidizes towards an increase in the abundance of denitrifying genes due to a greater nitrate substrate concentration, as both forms of N (ammonium N and nitrate N) have positive relationships with denitrifying gene abundances (Xiao et al., 2021). This could be the result of the stimulation of microbial growth and activity by improved nutrient availability, and improved soil physical properties can make the soil environment more suitable for microbial growth (Ai et al., 2012). Meanwhile, it was quite fascinating to find that the Nar enzyme activity of A1 decreased with an increase in the rate of N input, highlighting that this mixture could be the best choice to improve the soil nutrient balance, but the reason for this is unknown. However, a decreased Nir enzyme activity at N3 input could be attributed to the greater ammonium ion concentration at a high N input rate that resulted to starting the inhibition of Nir enzyme activity (Piotrowska and Wilczewski, 2012). Moreover, it is widely accepted that legumes are natural N fixers and contribute less to environmental pollution *via* ammonia volatilization or leaching—that is why these resulted in lower Nir activities compared with others.

### 4.2 Response of soil nutrients of different cropping systems to N input

Soil serves as the most important substrate for plant growth and development being a reservoir of many nutrients and a site for the microbial decomposition of plant and animal residues. Soil physical and chemical properties have a substantial influence on the plant community dynamics as a substrate for plant growth and development as well as a critical environmental factor (Li et al., 2022). In this study, the contents of ammonium N and nitrate N were significantly influenced by the N level and cropping system, but total N was not affected by them. The white clover and A1 had a significantly greater ammonium N content at the N2 and N3 inputs (no significance difference) compared with others, highlighting that the soil environment of these treatments allowed urease enzyme to convert urea into ammonium N, along with N addition as substrate for urease enzyme. However, ammonium N decreased or did not influence at a higher rate of N input, which might be because of the absorption of mineral N by soil microorganisms which suppressed the urease activity (Meysner et al., 2006). Nitrate N significantly increased with an increased rate of N input and A2 had the greater nitrate N content at N3 input compared with other cropping systems. This highlights that mixed sowing along with N input is beneficial to enhance the available N in the soil. Ammonium N is the most important substrate for ammonia-oxidizing microorganisms that contribute towards an increase in nitrate N via ammonia oxidation (Taylor et al., 2012), that is why nitrate N increased with increased N fertilization rate. However, the nitrate N content of alfalfa and white clover monocultures first increased and then decreased with an increase in the rate of N input, highlighting the N loss to environment at higher N rates.

### 4.3 Response of forage yield and nutritional quality of different cropping systems to N input

Forage yield is an important indicator to measure forage resources, which determines the amount of food provided by forage crops for livestock (Kawamura et al., 2008). A general concept prevails that N addition always leads to a greater forage yield. In this study, A1 and A2 had a greater forage yield than their respective monocultures under various N inputs. This result is consistent with the urease enzyme activity which played a crucial role to enhance the available nutrients for plant uptake—a strong positive correlation was found between forage yield and urease enzyme activity in this study. Moreover, the inclusion of legume in the mixture supplies more N to grasses by their N fixing ability, ultimately leading to the better growth and development of grasses (Tahir et al., 2022). In addition, the forage yield of mixtures increased up to a certain level with an increase in the rate of N input (especially up to the N2 threshold level), suggesting that higher N input rates are not beneficial and N can be lost to the environment.

Different planting patterns influence the forage yield and nutritional quality in grassland cultivation (Tahir et al., 2022). The nutritional quality of forage can not only directly affect the growth, reproduction, forage–herbivore interaction, and foraging behavior of livestock and wild herbivores by affecting the difficulty in obtaining nutrients but can also indirectly affect the yield, quality, and economic benefits of livestock products (Cui et al., 2016). From a nutritional value perspective, CP is an essential nutrient for livestock, and its content not only affects the economic benefits of forage but also directly affects the milk yield and milk protein yield of livestock (Yang et al., 2017). In this study, the cropping system significantly affected the nutritional quality parameters (CP, WSC, NDF, and ADF) while the nitrogen level just had a significant effect on CP, highlighting that the cropping system is more critical to control the nutritional quality of forages. The alfalfa and white clover had the greater CP content while having lower fiber contents (NDF and ADF) compared with other treatments, and the CP content slightly increased with an increase in N input, but the fiber contents were not affected. It is well established that legumes had a greater protein content and lower fiber contents compared with the grasses (Klupšaitė and Juodeikienė, 2015)—that is why the legumes were rich in protein content, and the grasses were abundant in fiber contents, and their mixtures were in between as there were negative correlations found between CP and fiber contents. Moreover, white clover had the greater WSC content, followed by A2 than the other treatments.

## 5 Conclusion

N addition and legume–grass mixed seeding significantly influenced the forage yield, nutritional quality, soil nutrients, and soil enzyme activities. The A1 mixture under N2 had a greater forage yield of 13.88 t ha^−1^ year^−1^ than the other N inputs with higher urease and HAO enzyme activities, which played a significant role to release more available N for plant uptake. Moreover, the A2 mixture under the N3 input had a greater forage yield of 14.39 t ha^−1^ year^−1^ than the N1 input with higher urease and Nar enzyme activities, but it was not substantially greater than the N2 input (13.80 t ha^−1^ year^−1^). Therefore, the growing of legume–grass mixtures under the N input of 300 kg ha^−1^ is recommended, which provides guidance for eco-friendly, sustainable, and cost-effective forage production in Sichuan, China.

## Data availability statement

The original contributions presented in the study are included in the article/supplementary material. Further inquiries can be directed to the corresponding author.

## Author contributions

MT: writing—original draft preparation, methodology, and software. XW: methodology, resources, and formal analysis. HL: investigation and data curation. JL: writing—review and editing and visualization. JZ: investigation and data curation. BK: validation and formal analysis. DJ: resources and validation. YY: project administration, funding acquisition, supervision, and investigation. All authors contributed to the article and approved the submitted version.
